# RISK FACTORS FOR DISENROLLMENT AMONG HOME HOSPICE PATIENTS WITH DEMENTIA

**DOI:** 10.1093/geroni/igz038.2911

**Published:** 2019-11-08

**Authors:** Elizabeth A Luth, David Russell

**Affiliations:** 1 Weill Cornell Medicine, New York, New York, United States; 2 Visiting Nurse Service of New York, New york, New York, United States

## Abstract

Hospice delivers care to a substantial and growing number of individuals with primary and comorbid dementia diagnoses. Dementia diagnosis and racial/ethnic minority status are risk factors for hospice disenrollment. However, little research examines racial/ethnic disparities and other risk factors for hospice disenrollment among hospice patients with dementia. This paper uses multinomial logistic regression to explore sociodemographic and functional status risk factors for hospice disenrollment among 3,949 home hospice recipients with primary or comorbid dementia. Results indicate that patients with a primary dementia diagnosis, racial/ethnic minority groups, and those higher functional status have elevated risk of disenrollment due to hospitalization, disqualification, and electively leaving hospice care. Additional research is needed to understand why primary dementia diagnosis and underrepresented racial/ethnic status are associated with multiple kinds of hospice disenrollment so that hospice practice can be tailored to respond to the needs of these individuals.

